# Establishment of a Practical Sperm Cryopreservation Pathway for the Axolotl (*Ambystoma mexicanum*): A Community-Level Approach to Germplasm Repository Development

**DOI:** 10.3390/ani14020206

**Published:** 2024-01-08

**Authors:** Nicholas Coxe, Yue Liu, Lucía Arregui, Rose Upton, Sarah Bodenstein, Steven Randal Voss, Maria T. Gutierrez-Wing, Terrence R. Tiersch

**Affiliations:** 1 Aquatic Germplasm and Genetic Resources Center, School of Renewable Natural Resources, Louisiana State University Agricultural Center, Baton Rouge, LA 70820, USAsboden2@lsu.edu (S.B.); mwing@agcenter.lsu.edu (M.T.G.-W.); 2 Louisiana Sea Grant College Program, Louisiana State University, Baton Rouge, LA 70803, USA; 3 Department of Neuroscience, Ambystoma Genetic Stock Center and Spinal Cord and Brain Injury Research Center, University of Kentucky, Lexington, KY 40536, USA

**Keywords:** axolotl, *Ambystoma*, sperm, cryopreservation, pathway, process map, repository

## Abstract

**Simple Summary:**

Amphibians are vitally important in biomedical research and conservation. Preserving germplasm materials (e.g., sperm, egg, embryos) in cryopreserved form can reduce the costs and risks associated with maintenance of live organisms. The goal of this work was to establish an initial practical sperm cryopreservation pathway for axolotls (*Ambystoma mexicanum*) to provide a foundation for development of germplasm repositories. The specific objectives were to (1) establish an efficient approach for sperm collection and quality evaluation; (2) evaluate the effects of osmotic pressure of extender solutions on sperm quality during refrigerated storage; (3) evaluate the effects of cryoprotectants on post-thaw sperm quality; (4) evaluate the feasibility of fertilization with thawed sperm; and (5) establish a cryopreservation pathway by integrating the findings into a comprehensive process flow map. With this approach, axolotl offspring were produced with cryopreserved sperm for the first time. Future research can build from this work with the goal of developing a germplasm repository based on community-level integration at the *Ambystoma* Genetic Stock Center.

**Abstract:**

The axolotl (*Ambystoma mexicanum*) draws great attention around the world for its importance as a biomedical research model, but housing and maintaining live animals is increasingly expensive and risky as new transgenic lines are developed. The goal of this work was to develop an initial practical pathway for sperm cryopreservation to support germplasm repository development. The present study assembled a pathway through the investigation of axolotl sperm collection by stripping, refrigerated storage in various osmotic pressures, cryopreservation in various cryoprotectants, and in vitro fertilization using thawed sperm. By the stripping of males, 25–800 µL of sperm fluid was collected at concentrations of 1.6 × 10^6^ to 8.9 × 10^7^ sperm/mL. Sperm remained motile for 5 d in Hanks’ Balanced Salt Solution (HBSS) at osmolalities of 100–600 mOsm/kg. Sperm cryopreserved in 0.25 mL French straws at 20 °C/min in a final concentration of 5% DMFA plus 200 mM trehalose and thawed at 25 °C for 15 s resulted in 52 ± 12% total post-thaw motility. In six in vitro fertilization trials, 20% of eggs tested with thawed sperm continued to develop to stage 7–8 after 24 h, and a third of those embryos (58) hatched. This work is the first report of successful production of axolotl offspring with cryopreserved sperm, providing a general framework for pathway development to establish *Ambystoma* germplasm repositories for future research and applications.

## 1. Introduction

The development of cryopreserved germplasm repositories for aquatic species is immensely valuable to support the urgent need for preservation and management of genetic resources for biomedical research, conservation of imperiled species, aquaculture, and wild fisheries. Research in cryopreservation for aquatic species has long focused on the development of species-specific protocols. However, most protocols are not advanced further to address the need for germplasm repositories. A change of thinking is needed to progress toward a more comprehensive and scalable approach that is driven through pathway development. A pathway comprises a series of integrated processes from gamete collection through fertilization, including quality management based on utility for practical applications. Cryopreservation pathways should be protocol-independent and provide a framework for medium- to high-throughput sample processing to support germplasm repositories at a community level.

The axolotl (*Ambystoma mexicanum*) is critically endangered in the wild [1], with severe population declines since the 2000s [2]. In laboratories, the axolotl has one of the deepest pedigrees among research animals [3]. For 150 years, laboratory stocks of axolotls have been used to investigate mechanisms of development, evolution, physiology, and neurology. They are highly prized as research and educational models because of their capability to regenerate many of their body parts, including limbs, the spinal cord, and brain tissues [4].

Axolotl research stocks are made available to scientists and educators through the *Ambystoma* Genetic Stock Center (AGSC) at the University of Kentucky (Lexington, KY, USA) which maintains a captive-bred population that was founded roughly 90 years ago [5]. The AGSC is charged to sustain this irreplaceable population, distribute living animals to users, and import new genetic stocks developed within the community. With the CRISPR-era yielding a growing number of transgenic and knockout lines [6], and with finite resources to maintain living animals, there is an urgent need to develop new genetic management strategies. Development of a cryopreservation pathway would provide a framework for the AGSC to preserve infrequently used stocks, thereby freeing resources for frequently used and new stocks. More generally, it would enable the AGSC to function as a comprehensive germplasm repository to manage, archive, and distribute stocks for the axolotl research community. Incorporating germplasm repository activities into an existing facility can be complicated over the short term as new activities can compete for limited resources (e.g., time, equipment, and personnel), often without increased support. It is therefore critically important to optimize a pathway at the outset that will improve the efficiency of operations over the long term.

Like most salamanders, the axolotl employs internal fertilization. In natural spawning, spermatophores (i.e., sperm packets) deposited by males are picked up by females within their cloaca. Previous studies that explored cryopreservation of sperm in spermatophores yielded > 50% sperm membrane integrity after thawing, but without successful fertilization [7,8]. Although sperm collection using spermatophores can minimize stress to animals, their complex structure poses challenges for process standardization such as sperm extraction, homogenization, and concentration adjustment. Collection of sperm and eggs from live *Ambystoma* salamanders is also possible using hormonal stimulation and stripping [9,10], but to our knowledge, there are no reports of successful fertilization by use of cryopreserved axolotl sperm.

The goal of this work was to initiate pathway development for axolotl sperm cryopreservation and the use of thawed sperm in in vitro fertilization (IVF). The specific objectives were to (1) establish an efficient approach for sperm collection and quality evaluation; (2) evaluate the effects of osmotic pressure of extender solutions on sperm quality during refrigerated storage; (3) evaluate the effects of cryoprotectants on post-thaw sperm quality; (4) evaluate the feasibility of fertilization with thawed sperm; and (5) establish an initial cryopreservation pathway by integrating the findings into a comprehensive process flow map. With the development of this practical method, axolotl larvae were for the first time produced by use of thawed sperm. This work provides a pathway-driven approach for axolotls, which can serve as a model for repository development in other amphibian and aquatic species. The pathway can also serve as a shared resource for cooperative community involvement by enabling the integration of future research and application efforts. Shared use of the pathway at the community level can also indicate areas for which future work is needed.

## 2. Materials and Methods

### 2.1. Animal Husbandry

Protocols for the use of animals in this study were reviewed and approved by the Louisiana State University Institutional Animal Care and Use Committee (Protocol A2021-25). Axolotls (RRID:AGSC_100A; RRID:AGSC_101-A) used in this study (24 males, 11 females) were 15 months to 3 years old and were obtained from the *Ambystoma* Genetic Stock Center (RRID:SCR_006372), Lexington, KY, USA; and the Warm Spring Fish Technology Center, Warm Springs, GA, USA. They were maintained for >12 months in recirculating water systems containing dechlorinated municipal water dosed with calcium chloride to a general hardness value of 140–250 ppm at the Aquatic Germplasm and Genetic Resources Center (AGGRC, aggrc.com, accessed on 18 December 2023), Baton Rouge, LA, USA. Target values for water quality parameters were 15–21 °C, pH 7.0–8.5, and 12 h light: 12 h dark photoperiod. Axolotls were individually fed once daily (Monday–Friday) with eight (about 0.5 g) pellets feed (Rangen axolotl diet, Aquatic Foods Inc., Fresno, CA, USA, Amazon.com, accessed on 18 December 2023). Water changes of 30–50% were performed daily Monday–Friday. Feeding and water change were not performed on Saturday and Sunday. Additional water quality parameters were monitored weekly and maintained within an acceptable range, including ammonia (0–1.0 mg/L), nitrites (0–0.8 mg/L), and nitrates (0–20 mg/L). Males had body weights of 92–179 g, and females had body weights of 112–169 g.

### 2.2. Establishment of a Sperm Collection Approach

Males were weighed and injected in the dorsal musculature with 2 international units per gram (IU/g) of body weight with human chorionic gonadotropin (hCG) (BioVendor Laboratory Medicine Inc., Lot # RU-82209-4, Ashville, NC, USA) diluted in phosphate buffered saline (PBS) (Gibco, Ref # 10010-031, Grand Island, NY, USA) at a concentration of 2 IU/µL. Animals were returned to their tanks and collected again after 18–43 h. Animals were anesthetized in 0.15% tricaine methanesulfonate (MS-222 Syncaine, Syndel, Ferndale, WA, USA) that was diluted with aquarium water and buffered to a pH of 6.5–7.5 with sodium hydroxide (1.0 N standardized solution, VWR Chemicals, Radnor, PA, USA) (Supplementary File S1). When animals lost righting reflex after being flipped upside-down (about 15–20 min), they were removed and placed on a firm, inclined surface.

**Figure 1:**
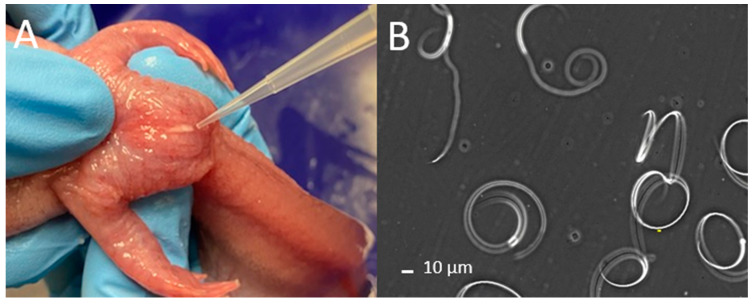
Collection and quality evaluation of axolotl sperm. (**A**) Stripping of a male axolotl by abdominal massage. (**B**) Sperm viewed with negative phase contrast microscopy (200× magnification). Companion videos of stripping and sperm motility can be found in the Supplementary Videos (Videos S1 and S2).

The abdomen and cloaca were rinsed with deionized (DI) water followed by Hanks’ Balanced Salt Solution at 200 mOsm/kg (HBSS200; pH 7.4) (Supplementary File S1). The sides and abdomen of the animals were massaged with gentle pressure from anterior to posterior toward the cloaca [9] (Figure 1A, Video S1). Massage motions were repeated, and sperm samples were collected using a micropipette (p1000 µL; Gilson Pipetman) (Figure 1A) and transferred to 1.5 mL centrifuge tubes. When necessary, larger animals were held with two hands when performing massages to better control pressure above the cloaca (Video S1). Urine was discharged with highly diluted sperm prior to the release of relatively concentrated sperm, and thus, the spermic urine (relatively clear liquid) and sperm fluid (opaque and white liquid) were collected in separate tubes. The time it took to anesthetize animals, collect sperm, and evaluate quality was recorded to identify potential bottlenecks during collection with limited personnel. The concentration (sperm/mL), volume (µL), and motility (described below) of collected sperm fluid were recorded, and the total number of sperm collected was calculated to evaluate relationships between animal size and sperm production.

### 2.3. Establishment of Quality Evaluation Approaches

Once collected, a 10 µL sperm sample was placed in a 1.5 mL centrifuge tube before the remaining sperm were diluted 1:1 with HBSS200 and placed on ice. In this work, pipette tips (Gilson Pipetman) were prepared by cutting off the distal 6 mm to minimize damage to sperm cells. To evaluate initial sperm concentration and motility, 10 µL samples were transferred to another centrifuge tube using a p10 micropipette and tips. Samples were diluted to a concentration ranging between 1 × 10^5^ and 5 × 10^5^ sperm/mL. This concentration range minimized sperm stacking to prevent estimation bias. A 5 µL sample was pipetted into a Neubauer-Improved Bright Line hemacytometer (Hausser Scientific, Buffalo, NY, USA). The average number of sperm within a 16-square grid (1 mm^2^) was calculated from five 16-square replicates. The number obtained from the hemacytometer was multiplied by the dilution factor to calculate the initial sperm concentration.

For motility, a 5 µL sample was pipetted onto a modified glass slide, which comprised a regular glass slide (75 mm × 25 mm) with the addition of two pieces of Magic^TM^ Tape (Scoth^®^, 3M Corporate, St. Paul, MN, USA) to create a 50 µm offset space between the base slide and the cover slip. The cover slip was placed on the tape and the samples were viewed by use of 200× magnification with a negative phase contrast microscope included in a computer-assisted sperm analysis (CASA) system (Hamilton Thorne Ceros II, Beverly, MA, USA). This CASA system can be substituted with a regular phase-contrast microscope. To assess motility, about 100 sperm cells per male were counted and classified into one of three classifications: spiraling, undulating, or static (Figure 1B, Video S2).

Spiraling sperm rotated in a circular pattern, undulating sperm had vibration along the undulating membrane of the sperm tails without spiraling movement, and static sperm were quiescent without spiraling or undulating membranes. It is unclear what specific roles spiraling and undulation could play during fertilization, so the percentage of total motility (spiraling plus undulating) was also calculated. Samples used to evaluate the effects of osmotic pressure of extender solutions on sperm quality were adjusted to a concentration of 1–5 × 10^5^ sperm/mL, and samples used to evaluate the effects of cryoprotectants on post-thaw sperm quality were adjusted to 2 × 10^6^ sperm/mL before freezing. Samples used to evaluate the feasibility of IVF with thawed sperm were adjusted to 1 × 10^6^ to 3.5 × 10^6^ sperm per mL before freezing.

### 2.4. Evaluation of Osmotic Pressure of Extender Solutions on Refrigerated Storage

To identify appropriate conditions of extender solutions to suspend axolotl sperm, the effects of various osmotic pressures of extenders were evaluated. Osmotic pressures of solutions were measured by use of a freezing point depression osmometer (Osmette III, Precision Systems^TM^, Basking Ridge, NJ, USA). Solutions with osmolalities of 100, 200, 300, 400, and 500 mOsm/kg were made by diluting HBSS600 with DI water followed by measurement. Undiluted sperm were collected, and samples were diluted in DI water (0 mOsm/kg, pH 8.3) and in HBSS solutions (pH 7.4) at six different osmolalities to produce a concentration of 1–5 × 10^5^ cells/mL. In six males, a 2 µL sample of sperm fluid was diluted at a ratio of 1:49 or 1:99, and in a single male, a 10 µL sample of spermic urine was diluted at a ratio of 1:9. After dilution, motility was estimated at 1, 3, 6, and 24 h, and when possible, at subsequent 24 h intervals for 6 d.

### 2.5. Evaluation of Cryoprotectants on Post-Thaw Sperm Quality

Sperm samples were collected and diluted to a concentration of ~2 × 10^6^ cells/mL with HBSS200 and held on ice. The choice of using HBSS at 200 mOsm/kg was made because that the osmolality matches that of sperm collected from the vas deferens (our unpublished data). Twelve cryoprotectant treatments were tested: 5 and 10% dimethylformamide (DMFA), dimethyl sulfoxide (DMSO), glycerol, and methanol; 5% DMFA plus 200 mM sucrose; 5% DMFA plus 200 mM trehalose; 5% DMSO plus 200 mM sucrose; and 5% DMSO plus 200 mM trehalose. Sperm suspensions were diluted 1:1 with each cryoprotectant solution, which was twice as concentrated before diluting.

Sperm were exposed with cryoprotectants with a 10 min equilibration time, defined as the duration from the addition of cryoprotectant to the initiation of cooling (marked by the decrease in chamber temperature below 4 °C) by use of a programmable freezer (IceCube 14M, SY-LAB, Purkersdorf, Austria). Cryoprotectant solutions were held at room temperature (22–24 °C) prior to mixing with sperm suspensions. Within the 10 min of equilibration time, a 100 to 200 µL sample of each cryoprotectant (12) and HBSS200 control (1) treatment was loaded individually by hand into a single 0.25 mL French straw (IMV Technologies, Maple Grove, MN, USA) and sealed using an ultrasonic straw sealer (ULTRASEAL21, Minitube, Delavan, WI, USA). Straws (13 per male) were placed into the freezer that maintained a chamber temperature of 4 °C prior to cooling. The program was started, and the freezer began to cool samples at a rate of 20 °C/min from 4 to −80 °C (chamber temperature). Motility of fresh sperm (without cryoprotectant) was estimated again. Frozen straws remained in the freezer for at least 1 min after the chamber temperature reached −80 °C, followed by transfer within 2 s to a Styrofoam box containing liquid nitrogen. Straws were loaded into 10 mm plastic visotubes (IMV Technologies, Maple Grove, MN, USA) attached to aluminum canes and transferred to a liquid nitrogen storage dewar (34HC Taylor-Wharton America Inc., Baytown, TX, USA). After 8–25 d, the straws were removed from the dewar, immediately thawed in a water bath at 25 °C for 15 s and transferred to 1.5 mL centrifuge tubes (Wards Sciences, Rochester, NY, USA). Thawed sperm were diluted with HBSS200 with a ratio of 1:9. The post-thaw motility was assessed at 2, 10, and 30 min after thawing.

### 2.6. Evaluation of the Feasibility of In Vitro Fertilization with Thawed Sperm

Fresh sperm concentration was adjusted to 9.2 × 10^5^ to 3.4 × 10^6^ sperm/mL (with HBSS200) and mixed with an equal volume of 10% DMFA with 400 mM trehalose (final concentration of 5% DMFA and 200 mM trehalose). This cryoprotectant was chosen given the results of the previous experiment. Samples were equilibrated at room temperature for 10 min, cooled at a rate of 20 °C/min (from 4 to −80 °C), stored for 2 d to 6 months in liquid nitrogen, and thawed at 25 °C for 15 s prior to fertilization experiments. Fresh sperm (adjusted to a concentration of 6.3 × 10^5^ to 1.0 × 10^6^ sperm/mL) were collected to assess the egg quality.

Females were weighed and injected in the dorsal musculature with 4 IU/g hCG diluted in PBS at a concentration of 4 IU/µL. Immediately after injection, females were placed in 80 L glass aquaria (filled aquarium water) at room temperature conditioned with a sponge filter connected to an air pump. To facilitate egg laying, 250 mm segments of airline tubing with a diameter of 3/16 inch (4.8 mm) were placed in the aquaria and weighted with air stones at each end. Females were held in the aquaria overnight and would typically begin to lay unfertilized eggs on the airline tubing within 24 h. Eggs used in IVF were collected within 10 min after deposition, placed into empty glass petri dishes (with a diameter of 8.5 cm and a height of 4.5 cm), and fertilized with either fresh or thawed sperm within 10 min of being placed in the petri dishes. A volume of 2–6 µL of sperm suspension was directly applied to each egg using a p10 micropipette and cut pipette tips. Thawed sperm were used for IVF within 5 min after thawing.

**Figure 2:**
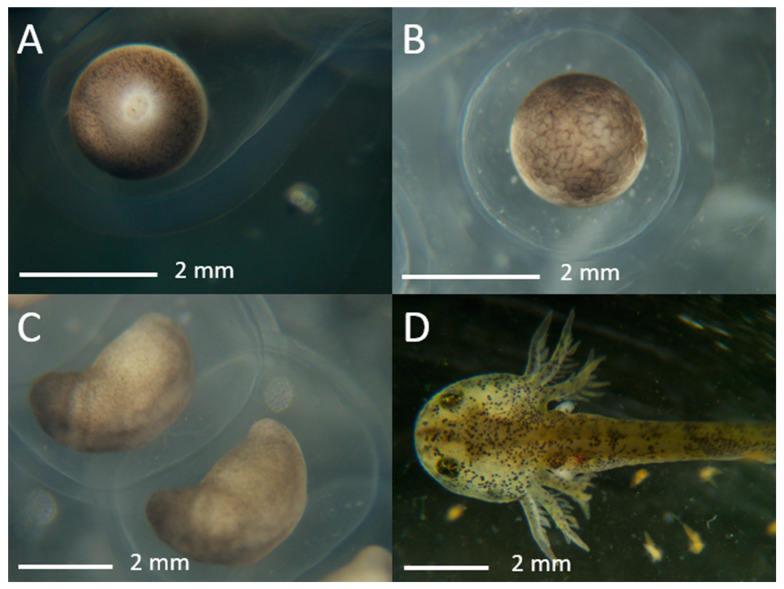
Evaluation of axolotl development. (**A**) Unfertilized or non-developing egg at 24 h. Developed embryos produced using thawed sperm at 24 h (**B**), 72 h (**C**), and upon hatching at about 15 d (**D**).

Fertilized eggs were left for 10 min prior to addition of 150–200 mL of 50% amphibian rearing water (ARW; 20% modified Holtfreter’s solution) with a osmolality 25 mOsm/kg [5] diluted with DI water to submerge the eggs. The 50% ARW was changed daily, and any non-developing embryos were removed after 24–48 h. The number of developing animals was counted at 24 and 72 h by use of a dissection stereoscope, and upon hatching (after about 15 d) (Figure 2). Typically developed embryos were at late morula stage (Stage 7–8) at 24 h and neurula stage (Stage 23) at 72 h. If embryos were not at these stages by these time points, they were classified as “not developing”. Embryos that reached hatching stage (Stage 43) but did not hatch were scored as dead.

### 2.7. Process Mapping

The steps of the axolotl sperm cryopreservation process were identified and defined during discussions with staff at the AGGRC. Defining of steps was accomplished by standardizing the tasks within each step, and establishing the beginning and end of each, as demarcated by certain tasks. Care was taken to avoid logical or temporal gaps in the process. The steps were outlined in a process flow diagram. The required wait time between steps and quality assurance decisions were also included in the diagram.

### 2.8. Statistical Analysis

Linear regression analyses were performed using data related to sperm collection (i.e., volume, concentration, body weight) and IVF (i.e., motility, concentration, percent developing embryos). Linear regression equations were reported if significant relationships were found. Data related to thawed motility were examined for normality and homogeneity of variance and analyzed using a single-factor repeated measures ANOVA followed by a pairwise *t*-test when significant differences were found. All analyses were performed using R 4.2.1 (R Foundation for Statistical Computing, Vienna, Austria, 2022). Figures were created using the R packages “ggplot2” [11] and “gridExtra” [12].

## 3. Results

### 3.1. Sperm Collection and Quality Evaluation

**Figure 3:**
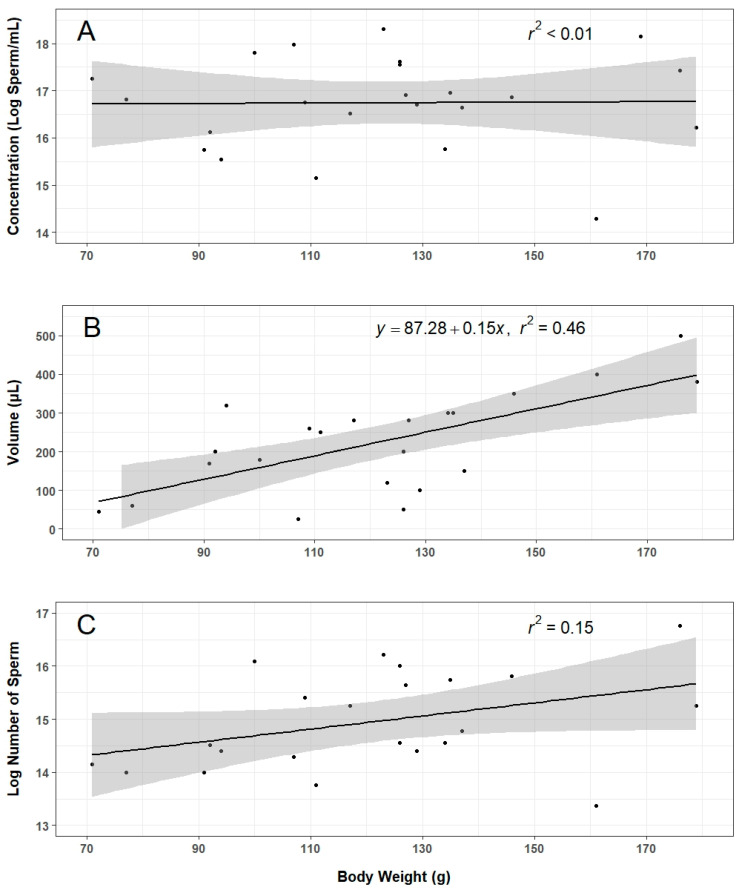
Relationships between the body weight of male axolotls and sperm concentration: ((**A**), n = 23), sperm volume ((**B**), n = 22), and total cell number ((**C**), n = 22) in sperm fluids collected by stripping. The shaded grey region indicates the 95% confidence level interval.

There were a total of 39 stripping attempts for sperm collection during this work, 37 of which were performed 18–24 h after hormonal injection. Two attempts were performed 42 and 43 h after hormonal injection due to limited time the previous day. Among all attempts, 28 produced sperm fluid or spermic urine at adequate volume and concentration to be used in the experiments. Nine attempts yielded inadequate volume or concentration to be aliquoted into experimental treatments, and two attempts yielded no sperm at all. Data on body weight, sperm volume, and concentration were recorded for 22 of the attempts in which samples were used in experiments (Figure 3). Collection of sperm from each male required about 30 min by experienced technicians starting when the animal was first placed in anesthetic to when the sperm sample was placed on ice and the animal was returned to a recovery tank. Two people were required to effectively strip an animal: one person to perform the stripping and the other to collect the sample with a pipette. Diluting a sperm sample (2 min) and assessing motility (5 min) and concentration (3 min) required an additional 10 min total with one person. If multiple animals were stripped in sequence, one person would assess motility and concentration while another anesthetized the next animal and reset the workspace.

There was considerable variation in sperm quantity and quality among males upon collection, with sperm volume ranging from 25 to 800 µL, concentration ranging from 1.6 × 10^6^ to 8.9 × 10^7^ sperm/mL, total sperm ranging 6.4 × 10^5^ to 1.9 × 10^7^ (Figure 3), and motility ranging from 7 to 60% for spiraling sperm, 25 to 74% for undulating sperm, and 11 to 38% for static sperm. There was a significant positive relationship between the animal body weight and sperm volume (Figure 3B, F-statistic = 17.1, DF = 20, *p* < 0.01), whereas no significant relationships were identified for body weight and concentration (Figure 3A) or total number of sperm (Figure 3C).

### 3.2. Refrigerated Storage

Sperm fluids were collected from seven males and used for the experiment. Upon collection and dilution in HBSS200, fresh samples showed 7–35% spiraling, 34–70% undulating, and 14–34% static sperm. In general, a 10% decline in total motility was found after 1 h of storage at 4 °C, and an additional 10% decline after another 2 h (Table 1). After 6 h of storage, samples maintained 50% of the initial total motility, which gradually declined, with some samples retaining 10% motility after 5 and 6 d of storage.

### 3.3. Cryopreservation and Post-Thaw Sperm Quality

**Figure 4:**
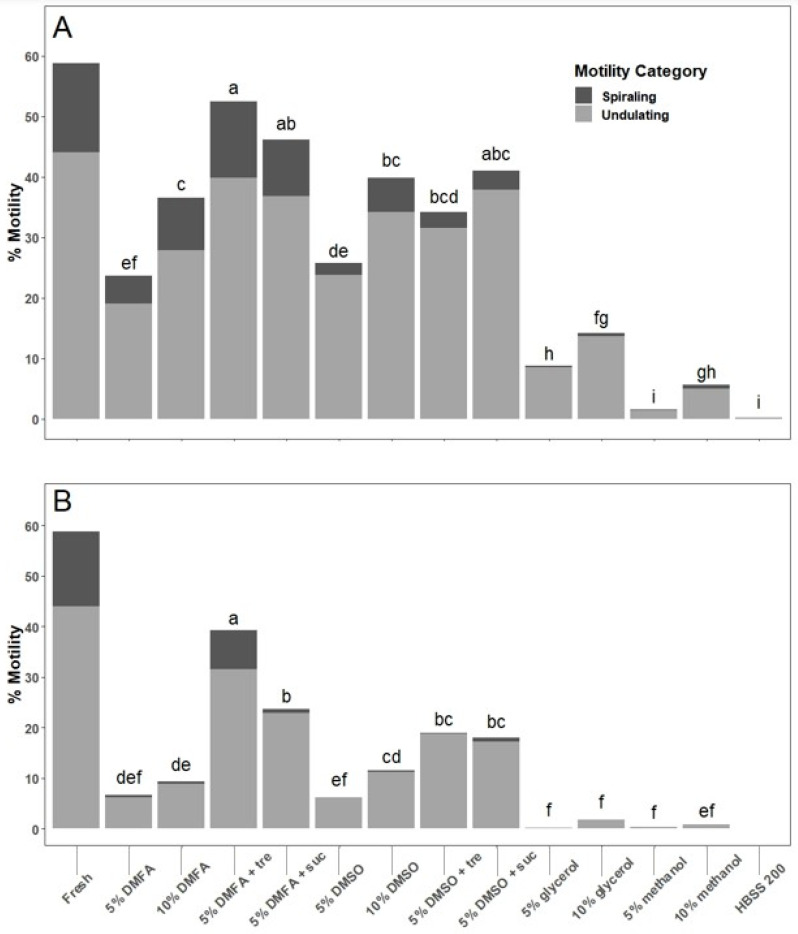
Motility of axolotl (n = 5) sperm prior to cryopreservation (fresh) and after thawing. Thawed sperm were equilibrated for 10 min in various cryoprotectant solutions, cooled at 20 °C per min to −80 °C, thawed at 25 °C for 15 s, and assessed at 2 min (**A**) and 10 min (**B**) after thawing. The final concentration of trehalose (tre) or sucrose (suc) used was 200 mM. Bars that do not share letters indicate significant differences in total motility (spiraling plus undulating) among groups. Fresh sperm were not included in the statistical analysis but were provided for reference.

Sperm fluids were collected from five males and used for the experiment. The percentage of total sperm motility at 2 min after thawing ranged from 0 to 52% among all cryoprotectants. Sperm cryopreserved and thawed in 5% DMFA plus 200 mM trehalose had higher total motility than all other cryoprotectants (*p* < 0.001), except for 5% DMFA plus sucrose and 5% DMSO plus sucrose (Figure 4). Total sperm motility at 10 min after thawing ranged from 0 to 39%. Sperm cryopreserved and thawed in 5% DMFA plus 200 mM trehalose had higher total motility than all other cryoprotectants (*p* < 0.001). From 10 to 30 min, the total motility decreased to 0% in all cryoprotectants.

### 3.4. In Vitro Fertilization with Cryopreserved Sperm

Eleven females were injected with hCG and laid between 25 and 700 eggs within 48 h. Eight females produced adequate eggs (≥50) within 24 h to be used in the experiments. Fresh sperm from four males and thawed sperm from nine males were used in IVF trials. Among all IVF trials, the use of fresh sperm resulted in 71 to 94% (Table 2) of eggs developing to Stage 7–8 at 24 h (Figure 2B). Thawed sperm also fertilized eggs with relatively higher variation, resulting in as high as 75% developing to Stage 7–8. Sperm samples from the same males and cryopreserved in the same batch (i.e., same date) tended to produce consistent percentages of developing embryos at 24 h, regardless of female or trial number. For example, using thawed sperm from Male 113 resulted in 45% developing embryos when paired with Female 17, 51% with Females 58 and 99, and 56% with Female 52.

**Figure 5:**
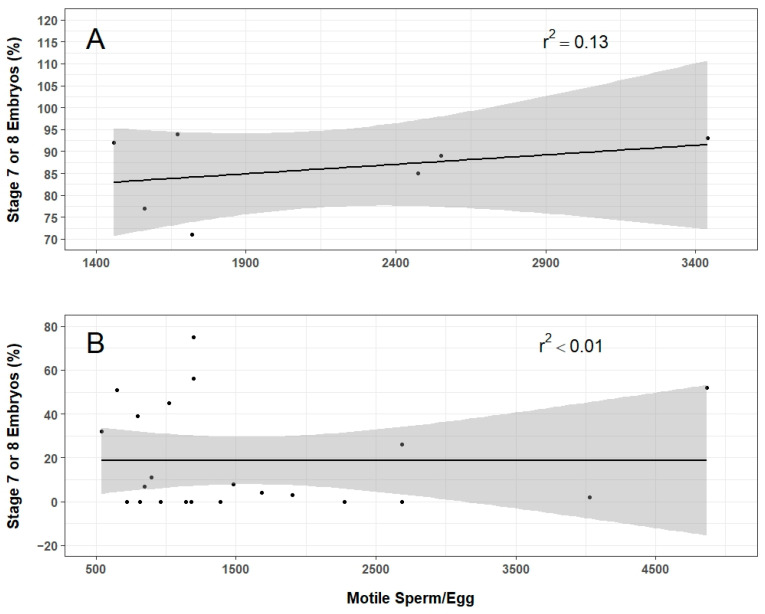
Relationships of the number of motile sperm per egg with the percentage of embryos reaching Stage 7–8 of development after 24 h when fresh sperm ((**A**), n = 7) and thawed sperm ((**B**), n = 22) were used. A linear regression and coefficient of determination (r^2^) was calculated for each group. The shaded grey region indicates the 95% confidence level interval.

Overall, 863 eggs were tested using thawed sperm, of which 20% developed to Stage 7 or 8. In total, a third of embryos that reached Stage 7 or 8 hatched, producing 58 animals. There was no significant relationship between total number of motile sperm per egg and percentage of embryos that developed to Stage 7–8 embryos (Figure 5).

### 3.5. Process Mapping

**Figure 6:**
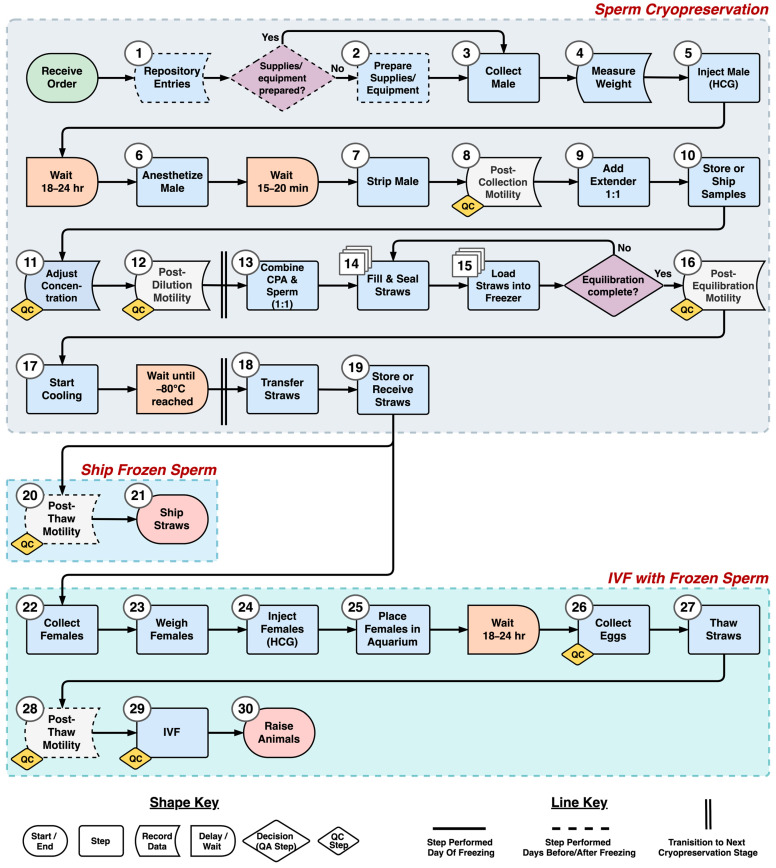
A process flow map [7] outlining a generalized pathway for axolotl sperm collection, cryopreservation, storage, egg collection, IVF, and raising of animals. There are also additional processes required for repository management, including shipping and receiving of samples and orders. Steps to assess motility (i.e., quality evaluation) were placed at critical points along the pathway. Quality control (QC) markers were placed on steps of the pathway where sperm should be evaluated to ensure quality and prevent wasted effort with further processing. The term “Equilibration” refers to the time elapsed from when sperm and cryoprotectant (CPA) are combined until straws begin cooling during cryopreservation. Equilibration occurs simultaneously as straws are filled with sperm sample and sealed. Steps denoted with stacked squares (Steps 14 and 15) could utilize different equipment options.

A process flow diagram was developed (Figure 6) based on interviews with staff members and the approaches developed. In total, 20 production steps, two quality assurance check points, and three significant wait times were identified for sperm collection and cryopreservation. An additional nine steps, two quality assurance check points, and one significant wait time were identified for egg collection and IVF. Five quality control steps, where either sperm motility or concentration should be measured and recorded, were also identified.

## 4. Discussion

Biomedical model stock centers that maintain large numbers of valuable animals are facing an increasingly challenging future. As more genetic lines are created and space becomes limited, the risks and expenses of holding many live animals in a single location will become unmanageable. The development of germplasm repositories will provide much needed relief of these problems for stock and resource centers such as the AGSC. For instance, the Zebrafish International Resource Center (ZIRC, Eugene, OR, USA), in partnership with the AGGRC, has developed comprehensive repository capabilities and has now shifted to maintaining more than 12,500 lines as frozen samples (zfin.org, accessed on 18 December 2023) in addition to actively used live populations. The work shown here outlined a practical pathway for axolotl sperm cryopreservation and demonstrated the feasibility for IVF by use of thawed sperm. This provides a framework for future research on optimization, standardization, and reproducibility towards medium- and high-throughput production at repositories.

The two common methods of noninvasive sperm collection for salamander species are the collection of spermatophores [13,14] and the collection of sperm fluid by stripping [9,15]. The collection of spermatophores and stripping have each been used in axolotls [7,9,14] and other *Ambystoma* species [15,16]. Each provides unique benefits and constraints that may affect the potential for medium- or high-throughput processing necessary for repository production and management. For example, the collection of spermatophores can minimize handling stress of the animals, but introduces complications in sperm quality evaluation. In addition, it requires increased need for labor and time, because mating behaviors need to be continuously monitored for hours to ensure timely collection of spermatophores prior to uptake by females. The stripping of axolotl sperm involves relatively more animal handling, including hormone injection, fasting (e.g., 1–3 d prior to collection), anesthetization, and massaging. Anecdotally, staff at the AGGRC have stripped more than ten males multiple times and have not observed negative effects on appearance or behavior, however the effect of stripping on long-term health requires investigation in future research. The benefits of stripping are the higher purity and volume of sperm samples, although hormonal injection was not always effective.

Roughly 70% of males injected with hCG produced sperm at sufficient volume and concentration to be used for this work. A “sufficient” amount of sperm was admittedly subjective given the experimental constraints, and future work should establish a minimum threshold to continue processing sperm samples. When adequate sperm were collected, the variation in volume and concentration was considerable. Although sperm volume was somewhat predicted by animal body weight, there was no significant relationship between concentration or total sperm count and body weight. Further investigations into the effects of factors such as animal age, weight, diet, time between stripping events, and stripping technique on sperm production will be essential to developing more consistent collection methods.

Another critical component of a repository operation is the establishment of quality management practices [17]. Common measures of sperm quality evaluation include motility, concentration, morphology, and membrane integrity [18]. The large size of axolotl sperm (among the largest of vertebrates) prevents the use of typical settings in CASA systems, and thus motility was classified without CASA in this work. Previously published reports classified salamander sperm motility into two categories: ‘motile’ (circular movement) or ‘not motile’ (undulating and static) [9], or ‘motile’ (circular plus undulating) and ‘non-motile’ (static) [15]. In the present study, motility was categorized into three classes. Large variations were observed in all three motility categories among different males upon collection. This may result from differences in age, phenotype, time of collection, or differences in individual fitness. If motility plays a large role in overall sperm quality, it would be useful to incorporate a quality control step after collection in which sperm that do not meet a motility threshold are discarded to conserve resources. Further research into the relationship between sperm motility and fertilization will provide critical insight into the development of a robust quality management system. Membrane integrity may be another important measure of quality [19] to support quality management for repositories.

Storage in an extender solution is a critical process to maintain sperm quality during transport and in preparation for cryopreservation. For externally fertilized aquatic species (e.g., most teleost fishes) extender solutions are usually prepared as isotonic to the plasma or seminal fluid to keep sperm from activating [20,21]. In axolotls, which are internally fertilized, sperm are motile upon collection [9].The osmolality of sperm suspension collected from the vas deferens was ~180 mOsm/kg (our unpublished data). As such, an osmolality level of 180 mOsm/kg can be considered as a candidate for ‘isotonic’. In the present study, axolotl sperm tolerated suspension across a wide range of osmotic pressures (i.e., 100–500 mOsm/kg) for several d. The internal fertilization mechanism of axolotls and other caudates may contribute to sustained motility across a wide range of osmotic environments.

Although the motility of sperm in this study did generally decrease with time, the rate of decrease was more gradual than in studies of tiger salamander (*Ambystoma tigrinum*) sperm suspended in different extenders and at different osmolalities [13,15]. For instance, the total motility for tiger salamander sperm maintained in 10% Holtfreter’s solution (~12 mOsm/kg) and 2% trehalose plus 0.2% bovine serum albumin decreased to around 10% after 24 h at 4 and 0 °C. These trends were similar to ours in regard to sperm stored in DI water. Sperm maintained in HBSS remained motile for longer, though admittedly, there were differences in pH between DI water (pH 8.3) and HBSS (pH 7.4) that could have influenced motility over time. While, in this case, osmolality likely influenced motility to a larger degree than pH, future studies should consider these factors.

The ability of axolotl sperm to remain motile for a relatively long duration may facilitate repository operations. For example, sperm from multiple males could be collected throughout a day (or possibly multiple days) and be cryopreserved later. In addition, shipping of fresh sperm on ice from field collection sites to central facilities for cryopreservation can be evaluated for conservation purposes. Because HBSS is a commonly used sperm extender for aquatic species, it was used for initial assessment in this work. Other extenders that are commonly used for amphibians such as Holtfreter’s, simplified amphibian Ringer’s (SAR) and Marc’s modified Ringer’s (MMR) could also maintain axolotl sperm motility for days (our unpublished data) and should be investigated further.

Trials of multiple cryoprotectant treatments showed that a final concentration of 5% DMFA plus 200 mM trehalose resulted in the highest thawed motility given the specific freezing and thawing conditions used: 10 min equilibration at room temperature, cooling at 20 °C per min, and thawing in a 25 °C water bath for 15 s. Although this cryoprotectant combination has proven effective, alternative cryoprotectants should not be excluded from consideration for future work, as specific conditions (e.g., container, volume, and cooling and warming methods) can interact and may change the outcomes of cryopreservation. It should be acknowledged that because this was a pilot study, motility evaluation was not performed after equilibration prior to cryopreservation, a critical quality control step to identify toxic effects of cryoprotectants on sperm [22]. This should be included in future studies and routine repository quality control programs. After thawing, however, motility of sperm held in 5% DMFA plus trehalose and 5% DMSO plus trehalose reduced motility by only 10–15% between 2 and 10 min at room temperature. These cryoprotectants may therefore provide a useful window of operation to use thawed sperm, especially if thawed samples were kept on ice prior to use.

Trehalose is often used in cryobiology applications as an additional non-permeating cryoprotectant because of its ability to disrupt ice crystallization, protect proteins and other biomolecules, stabilize cell membranes, and increase membrane permeability [23]. In this work, treatments with trehalose showed superior effects in post-thaw sperm quality. However, trehalose is relatively expensive (~USD 1000 for 500 g, Thermo Scientific Chemicals as of September 2023) compared to other non-permeating cryoprotectants (e.g., sucrose, ~USD 50 for 500 g, Thermo Scientific Chemicals as of September 2023). As such, repositories should evaluate the cost and benefit when considering the use of cryoprotectants and other supplies [24] given various workflows and scales of production.

The IVF experiments demonstrated the feasibility of producing axolotl offspring with thawed sperm. These results suggest that the pathway approach developed herein can be practically applied for freezing and storing of axolotl stocks at AGSC. It is unclear at present which variables related to sperm quality are most critical for fertilization as motility, concentration, and total motile sperm per egg varied widely in relation to the number of developing embryos produced in these experiments. It is possible that the motility assessment did not account for sperm morphology or speed of movement. In this, relatively slow motile sperm would be classified the same as fresh sperm that often spiral or undulate more vigorously. Additionally, the concentration of thawed sperm used might be too low to consistently fertilize eggs. The potential interaction of these two factors could result in thawed sperm samples with similar motility and concentration producing embryos at different rates. Future work will continue to explore these factors to advance reproducibility. Understanding how sperm quality and volume relate to fertilization and embryo development will be critical for achieving consistent and high-throughput processing.

The initial process flow diagram developed in this study outlined the step sequence and flow of materials and information for axolotl sperm cryopreservation. The pathway concept can provide a generalizable community-level roadmap for future work. In addition, the flow diagram can be used to collect necessary data to identify and eliminate bottlenecks and processing waste [25,26] through simulation modeling. In future studies, step definitions could be expanded to include the resources required to perform each step. This would include the necessary equipment and supplies and their initial and per-use costs, the number of operators necessary, and the time required to complete each step.

To evaluate in greater detail the resources needed to perform a process such as cryopreservation, as indicated above, a common industrial engineering tool, discrete-event simulation (DES) modeling, can be used. Discrete-event models simulate processes through time, using process flow diagrams as the basic structure in combination with data (e.g., time, quality, and cost) required to complete each step [27,28]. With this information, an existing facility such as AGSC could evaluate how to best utilize current resources to incorporate new repository activities. It will be important to also consider that cryopreservation is not the only process that must be incorporated when developing a germplasm repository. Maintaining a database and the storage of physical samples are also key processes that can be modeled [29], as well as interactions with user communities.

## 5. Conclusions

The development of cryopreserved germplasm repositories for axolotls and other aquatic species will support the urgent need for the preservation and management of genetic resources for biomedical research, conservation of imperiled species, aquaculture, and wild fisheries. The cryopreservation pathway outlined in this study provided a framework to enable scalable repository operation at practical levels. Combined with process flow mapping, this can advance future work to develop approaches that can accommodate the inevitable need for high-throughput sample processing. For the sake of adoption and modification towards community-level standardization, a preliminary protocol derived within this pathway is provided (Supplementary File S1). However, this protocol should not be considered as a final standard, but rather a starting point to develop other, more practical and effective methods for sample processing and quality management. It would be extremely useful and efficient if future advances in repository research and application within the *Ambystoma* community could be integrated into a common flow diagram and pathway. This would provide a forum for integration of community efforts and would lead to much-needed standardization of procedures and terminology, and harmonization of results, which are pervasive problems that characterize aquatic species cryopreservation. Such community-level activities can be further leveraged by development and utilization of open hardware [30] approaches using inexpensive, widely accessible devices that can be shared as digital files for user fabrication.

## Figures and Tables

**Table 1:** Mean percentage (±SD) spiraling, undulating, and total (spiraling plus undulating) motility of sperm in seven osmotic pressures, including deionized (DI) water (0 mOsm/kg) and Hanks’ Balanced Salt Solution (HBSS) through time. The sample sizes (n) of number of males are provided for each time point. A double dash “--” indicates samples that were discarded after all sperm were observed as static for more than 2 d.

				HBSS with Various Osmolalites (mOsm/kg)					
Category	Time	n	DI Water	100	200	300	400	500	600
Spiraling	1 h	7	25 ± 20	15 ± 9	17 ± 9	14 ± 9	13 ± 6	7 ± 4	6 ± 3
	3 h	6	20 ± 15	13 ± 11	12 ± 7	9 ± 8	13 ± 9	8 ± 7	5 ± 7
	6 h	5	14 ± 10	10 ± 7	11 ± 12	16 ± 11	18 ± 9	13 ± 10	7 ± 8
	1 d	7	3 ± 3	13 ± 6	13 ± 9	13 ± 9	16 ± 9	12 ± 10	6 ± 7
	2 d	7	0	10 ± 7	11 ± 8	11 ± 9	10 ± 7	11 ± 9	4 ± 2
	3 d	7	0	11 ± 7	14 ± 13	10 ± 10	7 ± 6	5 ± 4	3 ± 4
	4 d	5	--	12 ± 5	9 ± 8	8 ± 4	4 ± 1	3 ± 2	0
	5 d	3	--	9 ± 8	6 ± 7	4 ± 3	5 ± 3	4 ± 3	2 ± 1
	6 d	3	--	4 ± 3	4 ± 2	2 ± 3	4 ± 5	1 ± 2	0
Undulating	1 h	7	25 ± 14	44 ± 13	43 ± 14	38 ± 9	46 ± 11	39 ± 16	29 ± 12
	3 h	6	18 ± 9	34 ± 9	38 ± 10	31 ± 15	33 ± 13	33 ± 14	25 ± 13
	6 h	5	12 ± 5	35 ± 17	21 ± 6	20 ± 9	30 ± 12	30 ± 13	27 ± 12
	1 d	7	5 ± 4	12 ± 6	16 ± 8	17 ± 7	22 ± 14	22 ± 10	17 ± 10
	2 d	7	0	17 ± 5	16 ± 9	14 ± 10	22 ± 14	19 ± 9	19 ± 12
	3 d	7	0	14 ± 8	10 ± 7	12 ± 9	14 ± 11	12 ± 7	10 ± 9
	4 d	5	--	10 ± 10	5 ± 6	12 ± 6	17 ± 10	11 ± 4	7 ± 7
	5 d	3	--	4 ± 1	4 ± 3	3 ± 2	8 ± 7	8 ± 10	4 ± 4
	6 d	3	--	4 ± 2	6 ± 6	6 ± 2	7 ± 2	2 ± 3	0
Total Motility	1 h	7	50 ± 17	59 ± 10	59 ± 18	52 ± 15	59 ± 8	45 ± 18	35 ± 14
	3 h	6	38 ± 15	47 ± 18	50 ± 16	40 ± 18	46 ± 15	41 ± 11	29 ± 14
	6 h	5	26 ± 10	45 ± 16	32 ± 12	36 ± 18	48 ± 14	43 ± 16	34 ± 19
	1 d	7	7 ± 5	26 ± 9	29 ± 14	30 ± 14	38 ± 16	34 ± 17	23 ± 15
	2 d	7	0	28 ± 11	28 ± 15	25 ± 13	32 ± 15	30 ± 15	23 ± 12
	3 d	7	0	25 ± 14	24 ± 19	22 ± 16	22 ± 14	17 ± 9	13 ± 12
	4 d	5	--	22 ± 10	14 ± 12	21 ± 9	21 ± 10	14 ± 5	7 ± 7
	5 d	3	--	13 ± 7	10 ± 9	7 ± 6	13 ± 8	12 ± 13	6 ± 7
	6 d	3	--	8 ± 2	10 ± 7	8 ± 5	12 ± 6	3 ± 5	0

**Table 2:** Summary of in vitro fertilization (IVF) trials using fresh and thawed axolotl sperm. Horizontal lines separate individual trials that were conducted on the same day (d). The percentages (%) of developing or hatched embryos based on the number (#) of fertilized eggs are reported for each observed time point. Males that were stripped a second time on a different date are denoted as “_2”. Eggs from one or two females were used for each sperm sample in each trial. A double dash “--” indicates missing data points.

	Male ID	Sperm Condition	Female ID	Concentration (Sperm/mL × 10^5^)	% Total Motility	Volume Per Egg (µL)	Motile Sperm Per Egg	# Eggs	% Stage 7–8 (24 h)	% Stage 23 (72 h)	% Hatched (12–15 d)
Trial 1	114	fresh	17	10.0	85	2–4	1700–3400	100	89	64	--
	38	thawed	17	5.0	53	2–4	530–1060	18	39	22	--
	39	thawed	17	15.0	--	2–4	--	38	40	0	--
	113	thawed	17	5.0	68	2–4	680–1360	141	45	7	--
Trial 2	37	fresh	98	10.0	73	2	1460	50	92	--	84
	104	thawed	98	7.0	58	2	812	31	0	0	0
	68	thawed	98	8.0	56	2	896	38	11	11	11
	28	thawed	98	8.7	68	2	1183	39	0	0	0
Trial 3	27	fresh	101	9.5	88	2	1672	17	94	--	--
	83	thawed	101	10	57	2	1140	29	0	0	0
	73	thawed	101	5.3	68	2	721	26	0	0	0
	68	thawed	101	8.0	53	2	848	30	7	7	7
Trial 4	37	fresh	58, 99	10.0	86	2	1720	31	71	--	--
	37	fresh	58	10.0	86	4	3440	14	93	--	--
	38	thawed	58, 99	5.0	54	2	540	44	32	--	14
	113	thawed	58, 99	5.0	65	2	650	43	51	--	44
	36	thawed	58, 99	11.0	61	6	4026	47	2	--	0
	36	thawed	99	17.0	67	2	2278	33	0	0	0
Trial 5	35	fresh	3, 115	6.3	62	4	1562	30	77	73	63
	113	thawed	115	4.6	65	4	1196	4	75	75	75
	68_2	thawed	3, 115	5.4	78	4	1685	47	4	4	4
	113_2	thawed	3, 115	5.6	43	4	963	72	0	0	0
	39_2	thawed	3, 115	6.2	56	4	1389	20	0	0	0
	113_2	thawed	3, 115	7.4	50	4	1480	13	8	0	0
	28_2	thawed	3, 115	8.8	54	4	1901	38	3	3	0
	38_2	thawed	3, 115	9.6	70	4	2688	66	26	17	11
Trial 6	114_2	fresh	52	6.8	91	4	2475	39	85	85	69
	113	thawed	52	4.6	65	4	1196	9	56	56	56
	38_2	thawed	52	9.6	70	4	2688	8	0	0	0
	38_2	thawed	52	16.0	76	4	4864	29	52	41	34

## Data Availability

Data are contained within the article and Supplementary Materials.

## Supplementary Materials

The following supporting information can be downloaded at: https://www.mdpi.com/article/10.3390/ani14020206/s1, File S1: Axolotl sperm cryopreservation protocol; Video S1: Stripping sperm from an anesthetized male axolotl via abdominal massage. Sperm fluid or spermic urine was collected with a p1000 pipette and transferred into 1.5-mL centrifuge tubes on ice.; Video S2: Freshly collected axolotl sperm viewed by use of negative phase contrast (200× magnification). Three classifications of motility are observed and labeled.
