# Supplementary material for: Establishment of a Practical Sperm Cryopreservation Pathway for the Axolotl (Ambystoma mexicanum): A Community-Level Approach to Germplasm Repository Development
Source: Animals (Basel). 2024 Jan 8;14(2):206. doi: 10.3390/ani14020206 (PMC10812443; doi:10.3390/ani14020206)
Supplement: Supplementary file 1 [file animals-14-00206-s001.zip › Supplimental File S1.pdf]

Axolotl Sperm Cryopreservation Protocol

Pathway summary: Sperm Collection (PR-01 and PR-02), Sperm Refrigerated Storage (PR-03), Sperm Cryopreservation (PR-04), Egg Collection (PR-05 and PR-06), Sperm Thawing (PR-07), *In-vitro* Fertilization and Embryo Care (PR-08)

PR-01: Hormonal Injection (male)

1. Select and weigh male.
2. Inject male intramuscularly and dorsally with 2 IU of hCG per g of body weight.
3. Return male to tank or aquarium.

PR-02: Sperm Collection via Stripping

1. Wait 18-24 h after hormonal injection.
2. Submerge male in 0.15% MS-222 solution.
3. Wait until loss of righting reflex, or about 15-20 min.
4. Remove male from MS-222 solution and place on a firm, flat surface.
5. Rinse abdomen and cloaca with deionized (DI) water, followed by HBSS200.
6. Perform abdominal massage on male by applying pressure down the abdomen from anterior to posterior to the cloaca. Larger animals can be held with two hands instead of resting on a flat surface. Repeat this motion until spermic urine and milt are discharged.
7. Collect spermic urine and milt using a p1000 pipette and transfer both into separate centrifuge tubes on ice.
8. *Quality Evaluation 1:* Dilute sperm sample in extender to evaluate motility and concentration.
9. Combine extender and sperm at a ratio of 1:1 and homogenize by flicking and/or inverting tube.

PR-03: Sperm Refrigerated Storage

1. Store sperm in centrifuge tubes in refrigerator (4 °C) or on ice (0 °C) if desired.

PR-04: Sperm Cryopreservation

1. If necessary, label straws before use.
2. *Quality Evaluation 2:* Dilute sperm sample in extender to evaluate motility and concentration.
3. Adjust sperm to desired concentration for cryopreservation (≥ $2x10^6$ sperm/mL) while keeping sperm on ice.
4. Remove cryoprotectant (CPA) from refrigerated storage and warm to 22-24 °C.
5. Remove sperm from ice and combine with CPA at a ratio of 1:1. *This step marks the start of equilibration.*
6. While sperm and CPA equilibrate at 22-24 °C, load mixture into straws and seal.
7. After 10 min of equilibration, load straws into a freezer (4 °C chamber temperature).
8. Begin cooling to -80 °C. *This step marks the end of equilibration.*
9. *Quality Evaluation 3:* After equilibration has ended, evaluate motility.
10. Once freezer chamber has reached -80 °C, wait ≥ 5 min before removing straws to ensure -80 °C has been reached inside the straws.
11. Within 2 s, transfer straws from freezer to liquid nitrogen (LN) transfer container.

12. Under the LN surface, load straws into viso tubes and aluminum canes in preparation for storage.
13. Within 2 s, transfer canes from LN transfer container to LN storage container.

PR-05: Hormonal Injection (female)

1. Select and weigh female.
2. Inject female intramuscularly and dorsally with 4 IU of hCG per g of body weight.
3. Place female in tank or aquarium with aerated water and segments of 5-mm rubber airline tubing weighted down with air stones.

PR-06: Egg Collection

1. Wait 18-24 h after hormonal injection. Females will usually begin laying within this time frame.
2. Remove eggs that were laid > 10 min after deposition, or if time of deposition is unknown.
3. Collect eggs within 10 min of deposition.
4. Transfer eggs to fertilization container. Glass dishes work well. Eggs can be kept on airline tubing or removed with scissors or a scalpel before transferring to fertilization container.

PR-07: Sperm Thawing

1. Within 2 s, transfer canes from LN storage container to transfer LN container.
2. Under the LN surface, remove straws from viso tubes.
3. Within 2 s, transfer straws to 25 ℃ water bath and stir under the water surface for 15 s.
4. After 15 s, remove straws from water bath and dry.
5. Cut the sealed side of the straw with scissors and place inside a centrifuge tube on ice.
6. Cut the cotton end of the straw and push liquid sample out using a 1-mL syringe with cut pipette tip.
7. *Quality Evaluation 4:* Evaluate motility and concentration within 2 min of thawing.

PR-08: In-vitro Fertilization and Embryo Care

1. Within 10 min of eggs being collected from water, and 5 min within sperm being thawed, apply sperm directly to eggs using a p10 or p20 pipette. It is recommended to use ≥ 2 μL per egg with a concentration of ≥ $2x10^6$ sperm/mL.
2. Allow sperm and eggs to sit exposed to air for 10 min.
3. Fill fertilization container with 50% axolotl rearing water (ARW) so that eggs are fully submerged.
4. Change 50% ARW daily.
5. Assess embryo development after 24 (Stage 7-8) and 72 h (~Stage 23), and upon hatching.

Solution Recipes and Instructions

Hanks' balanced salt solution 200 mOsm/kg (HBSS200)

- Deionized (DI) water
- NaCl (5.36 g/L)
- $CaCl_2.2H_2O$ (0.11 g/L)
- KCl (0.27 g/L)
- $MgSO_4.7H_2O$ (0.13 g/L)
- KH_2PO_4 (0.04 g/L)
- Na_2HPO_4 (0.04 g/L)
- Glucose (0.67 g/L)
- $NaHCO_3$ (0.23 g/L)

1. Combine ingredients one at a time and increase to the final volume with DI water

10% dimethylformamide (DMFA) plus 400mM trehalose dihydrate

- 1M HEPE-NaOH solution (1 mL/50 mL water)
- 99% pure trehalose dihydrate (7.56g/50 mL water)
- DMFA (5 mL/50 mL water)

1. Combine 1 mL HEPE-NAOH solution with 49 mL nano pure water
   - Makes 20 mM HEPE-NAOH solution
2. Combine 7.56 g trehalose dihydrate with 50 mL 20 mM HEPE-NaOH solution
   - Makes 400 mM trehalose dihydrate solution
3. Combine 5 mL DMFA with 45 mL 400 mM trehalose dihydrate solution
   - Makes 10% DMFA in 400 mM trehalose dihydrate solution (pH 7.4)

50% axolotl rearing water (ARW)

- Deionized (DI) water
- NaCl (0.69 g/L)
- $CaCl_2.2H_2O$ (0.015 g/L)
- KCl (0.02 g/L)
- $MgSO_4.7H_2O$ (0.08 g/L)
- $NaHCO_3$ (0.04 g/L)

1. Combine ingredients one at a time and increase to the final volume with DI water
   - It may help to mix a stock solution (5-10X concentration) and dilute accordingly with DI water
